# Protocol for synthesis of di- and tri-substituted s-triazine derivatives

**DOI:** 10.1016/j.mex.2020.100825

**Published:** 2020-02-20

**Authors:** Ayman El-Faham, Anamika Sharma, Muhammad Farooq, Zainab Almarhoon, Rakia Abd Alhameed, Mohammad A.M. Wadaan, Beatriz G. de la Torre, Fernando Albericio

**Affiliations:** a Department of Chemistry, College of Science, King Saud University, P.O. Box 2455, Riyadh 11451, Saudi Arabia; b Department of Chemistry, Faculty of Science, Alexandria University, P.O. Box 426, Alexandria 21321, Egypt; c School of Chemistry and Physics, University of KwaZulu-Natal, University Road, Westville, Durban 4001, South Africa; d Department of Zoology, Bioproducts Research Chair, College of Science, King Saud University, P.O. Box 2455, Riyadh 11451, Saudi Arabia; e KRISP, College of Health Sciences, University of KwaZulu-Natal, Westville, Durban 4001, South Africa; f Department of Organic Chemistry, CIBER-BBN, Networking Centre on Bioengineering, Biomaterials and Nanomedicine, University of Barcelona, Martí i Franqués 1-11, Barcelona 08028, Spain

**Keywords:** Orthogonal chemoselectivity, Cyanuric chloride, Nucleophilic substitution, Amines, Alcohol

## Abstract

The present protocol describes the synthesis of di and tri-substituted s-triazine derivatives

• *s*-Triazine undergoes sequential nucleophilic substitution reaction but order of nucleophile is very crucial.
• It is very difficult to substitute any nucleophile except amine once amine is incorporated onto s-triazine.
• During the synthesis of *O,N*-type substituted s-triazine, always O-type should be incorporated first.

## Graphical abstract

**Figure:**
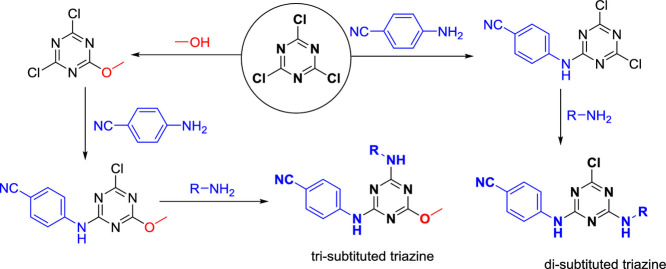

**Table:** Specification Table

Subject Area:	*• Chemistry More specific* • *Orthogonal chemoselective*
Method name:	*Sequential nucleophilic substitution*
Name and reference of original method:	*N/A*
Resource availability:	N/A

## Method details

Method details involves synthesis of di- and tri- substituted s-triazine derivatives as drawn in below scheme.

### Synthesis of di-substituted s-triazine using two sequential amines

*Step 1: Synthesis of 4-[(4,6-dichloro-1,3,5-triazin-2-yl)amino]benzonitrile*

### Materials required

• Cyanuric chloride
• Potassium carbonate (K_2_CO_3_)
• 4-Aminobenzonitrile
• Solvent [acetone, methanol (MeOH) and chloroform]
• Crushed ice and distilled H_2_O
• Rotary evaporator
• Glassware (Round bottom flask, beakers and conical flask)
• Silica-gel coated Aluminum TLC plates

### Procedure

*Note:* Cyanuric chloride is fuming solid. So, care must be taken to weigh it. Bottle must be opened in the fume hood with exhaust on.

(1) Cyanuric chloride (10 mmol) and 4-aminobenzonitrile (10 mmol) were dissolved in acetone (50 mL each) separately in conical flask.
(2) Both solutions were cooled to 0 °C.
(3) K_2_CO_3_ (10 mmol) was added to cyanuric chloride solution while stirring vigorously at 0 °C in a round bottom flask.
(4) A cold solution of 4-aminobenzonitrile was added dropwise to the stirring solution of cyanuric chloride and K_2_CO_3_.
(5) The reaction was stirred for 4 h at 0 °C (Note: maintaining temperature at 0 °C is very crucial for the reaction to avoid double incorporation).
(6) Monitor the reaction by TLC using 20% MeOH in chloroform as mobile phase in a closed system.
(7) Once no starting material appears on TLC, pour the reaction mixture onto crushed ice (1 L) in a beaker.
(8) Filter the solid product with distilled H_2_O (3 × 500 mL) and dry under high vacuum to obtain pure product.

*Note*: The current procedure is applicable for all amines (In case of aromatic amines the time consumed is 4 h whereas in case of aliphatic amine it only requires 30 min)

*Step 2: Synthesis of 4-[4-chloro-6-substituted(1,3,5-triazin-2-yl)amino]benzonitrile*

### Materials required

• K_2_CO_3_
• Piperidine
• Morpholine
• *N,N*’-Diethylamine
• Solvents [THF, ethyl acetate (EtOAc), hexane]
• Ice cold water and crushed ice
• Rotary evaporator
• Glassware (Round bottom flask, beakers and conical flask)
• Silica-gel coated Aluminum TLC plates

### Procedure

(1) 4,6-Dichloro (1,3,5-triazin-2-yl) aminobenzonitrile (10 mmol) and respective amine (piperidine, morpholine and diethyl amine) were dissolved in THF (50 mL each) separately in conical flask.
(2) K_2_CO_3_ (10 mmol) was added to 4,6-dichloro (1,3,5-triazin-2-yl) aminobenzonitrile solution while stirring vigorously at rt in a round bottom flask.
(3) Solution of respective amine was added dropwise to the stirring solution of 4,6-dichloro (1,3,5-triazin-2-yl) aminobenzonitrile and K_2_CO_3_.
(4) The reaction was stirred for 24 h at rt.
(5) Monitor the reaction by TLC using EtOAc-hexane (6:4) in a closed system.
(6) Once no starting material appears on TLC, THF was removed using rotary evaporator.
(7) Remaining reaction mixture was poured onto crushed ice (1 L) in a beaker.
(8) Filter the solid product with distilled H_2_O (3 × 500 mL) and dry under high vacuum to obtain product.
(9) The crude was recrystallized from EtOAc.

### Synthesis of trisubstituted triazines containing one alkoxy substituent and two amino substituents [1]

*Step 1: Synthesis of 2,4-dichloro-6-methoxy-1,3,5-triazine* [2]

### Materials required

• Cyanuric chloride
• Sodium bicarbonate (NaHCO_3_)
• Solvents (MeOH, EtOAc, hexane)
• Crushed ice and distilled H_2_O
• Rotary evaporator
• Glassware (Round bottom flask, beakers and conical flask)
• Silica-gel coated Aluminum TLC plates

### Procedure

*Note:* Cyanuric chloride is fuming solid. So, care must be taken to weigh it. Bottle must be opened in the fume hood with exhaust on.

(1) NaHCO_3_ (10 mmol) was dissolved in water and cooled to 0 °C.
(2) MeOH (50 mL) is added to the above solution and stirred vigorously at 0 °C.
(3) Cyanuric chloride (10 mmol) was added to the above stirring solution.
(4) The reaction was stirred for 3 h at 0 °C (Note: maintaining temperature at 0 °C is very crucial for the reaction to avoid double incorporation).
(5) Monitor the reaction by TLC using EtOAc-hexane (6:4) in a closed system.
(6) Once no starting material appears on TLC, excess of MeOH was removed under rotary evaporator.
(7) The residue was poured onto crushed ice (1 L) in a beaker.
(8) Filter the solid product with distilled H_2_O (3 × 500 mL) and dry under high vacuum to obtain pure product.

*Step 2: Synthesis of 4-chloro-6-methoxy(1,3,5-triazin-2-yl)amino)benzonitrile*

### Materials required

• NaHCO_3_
• 4-Aminobenzonitrile
• Solvents (acetone, EtOAc, hexane)
• Crushed ice and distilled H_2_O
• Rotary evaporator
• Glassware (Round bottom flask, beakers and conical flask)
• Silica-gel coated Aluminum TLC plates

### Procedure

(1) 2,4-dichloro-6-methoxy-1,3,5-triazine (10 mmol) and 4-aminobenzonitrile (10 mmol) were dissolved in acetone (50 mL each) separately in conical flask.
(2) NaHCO_3_ (1.38 g) was added to 2,4-dichloro-6-methoxy-1,3,5-triazine solution while stirring vigorously at 0 °C in a round bottom flask.
(3) Solution of 4-aminobenzonitrile was added dropwise to the stirring solution of 2,4-dichloro-6-methoxy-1,3,5-triazine and NaHCO_3_.
(4) The reaction was stirred for 24 h at rt.
(5) Monitor the reaction by TLC using EtOAc-hexane (6:4) in a closed system.
(6) Once no starting material appears on TLC, acetone was removed using rotary evaporator.
(7) Remaining reaction mixture was poured onto crushed ice (1 L) in a beaker.
(8) Filter the solid product with distilled H_2_O (3 × 500 mL) and dry under high vacuum to obtain product.

*Step 3: Synthesis of 4-substituted-6-methoxy((1,3,5-triazin-2-yl) aminobenzonitrile*

### Materials required

• K_2_CO_3_
• Piperidine
• Morpholine
• Pyrrolidine
• 4-Methylpiparizine
• 2-Hydroxyethylamine
• *N,N*’-Diethylaniline
• 4-Bromoaniline
• 4-Methoxyaniline
• Aniline
• Solvents [acetonitrile, EtOAc, ethanol (EtOH), hexane]
• Crushed ice and distilled H_2_O
• Rotary evaporator
• Glassware (Round bottom flask, beakers and conical flask)
• Silica-gel coated Aluminum TLC plates

### Procedure

(1) 4-chloro-6-methoxy(1,3,5-triazin-2-yl)amino)benzonitrile (10 mmol) and 4-aminobenzonitrile (10 mmol) were dissolved in acetonitrile (50 mL each) separately in conical flask.
(2) K_2_CO_3_ (10 mmol) was added to 4-chloro-6-methoxy(1,3,5-triazin-2-yl)amino)benzonitrile solution while stirring vigorously at rt in a round bottom flask.
(3) Solution of respective amine (piperidine, morpholine, pyrrolidine, *N*-methyl piperazine, 1-amino ethanol, *N,N*’-diethylamine, aniline, 4-bromoaniline and 4-methoxyaniline) dissolved in 10 mL acetonitrile was added to the stirring solution of 4-chloro-6-methoxy(1,3,5-triazin-2-yl)amino)benzonitrile and K_2_CO_3_.
(4) The reaction was refluxed for 18 h in an oil bath.
(5) Monitor the reaction by TLC using EtOAc-hexane (6:4) in a closed system.
(6) Once no starting material appears on TLC, acetonitrile was removed using rotary evaporator.
(7) Remaining reaction mixture was poured onto crushed ice (1 L) in a beaker.
(8) Filter the solid product with distilled H_2_O (3 × 500 mL) and dry under high vacuum to obtain product.
(9) The crude was recrystallized from 3:1 EtOAc-EtOH solvent mixture.

## Method validation

All the compounds were obtained in high yields and high purity as confirmed by ^1^H-NMR and ^13^C-NMR.
